# Antibiotic prophylaxis in elective laparoscopic cholecystectomy: a narrative review of efficacy, safety, and antimicrobial stewardship

**DOI:** 10.1097/MS9.0000000000004059

**Published:** 2025-10-13

**Authors:** Muhammad Umer Suleman, Muhammad Mursaleen, Umer Khalil, Muhammad Ali Subhani, Syeda Nafisa Tabassum

**Affiliations:** a Ayub Medical College, Abbottabad, Pakistan; b Department of Internal Medicine, Ayub Medical College, Abbottabad, Pakistan; c Department of Microbiology, Bangladesh Rural Advancement Committee, Dhaka, Bangladesh

**Keywords:** antibiotic prophylaxis, antimicrobial stewardship, elective surgery, evidence-based surgery, laparoscopic cholecystectomy, perioperative care

## Abstract

Elective laparoscopic cholecystectomy (LC) is associated with a low but measurable risk of surgical-site infection (SSI), with rates of 1–3% when antibiotics are withheld. This review evaluates randomized controlled trials, cohort studies, and meta-analyses published through June 2024 to assess the benefits and risks of perioperative antibiotic prophylaxis in low-risk patients. Evidence indicates that single- or multiple-dose regimens, most commonly cefazolin, reduce superficial SSI by 33–39% (relative risk 0.61–0.67; *P* < 0.01), equating to absolute risk reductions of 1.5–2.0% and numbers needed to treat between 30 and 50. There is no clear effect on deep or organ-space infections, which remain exceedingly rare. Antibiotic use carries potential harms, including *Clostridioides difficile* infection, allergic reactions, and the promotion of antimicrobial resistance. Current guidelines from leading surgical and infectious-disease societies recommend against routine prophylaxis in healthy individuals undergoing elective procedures to support antimicrobial stewardship. While selective prophylaxis may be warranted for patients with identifiable risk factors such as intraoperative bile spillage or significant comorbidities, routine administration in otherwise low-risk elective LC is not justified by the balance of evidence.

## Introduction

Elective laparoscopic cholecystectomy (LC) is generally a clean surgical procedure with a low overall rate of surgical-site infection (SSI). Indeed, large series report SSI rates of ~1–3% when no antibiotics are used^[1,2]^. Traditional surgical guidelines [e.g., Scottish Intercollegiate Guidelines Network (SIGN) and American Society of Health-System Pharmacists (ASHP)] do not recommend routine antibiotic prophylaxis for low-risk LC^[2,3]^, on the grounds that laparoscopic access minimizes infection risk. However, actual practice varies widely: for example, a UK cohort found ~61% of elective cases received prophylaxis despite the low baseline SSI rate^[4]^. Recent studies and meta-analyses have reexamined this controversy, with some suggesting a modest benefit to prophylaxis and others finding no clear effect. This article reviews current evidence and guidelines to weigh the benefits and drawbacks of routine perioperative antibiotics in LC. In this review, we stratify patients undergoing elective LC into three risk categories by integrating the American Society of Anesthesiologists (ASA) Physical Status Classification, WHO BMI ranges, and well-established surgical risk factors such as age, comorbidity burden, and intraoperative findings. The ASA system, widely used to communicate preoperative fitness, defines Class I as a healthy person and Class II as mild systemic disease, whereas Class III denotes severe systemic disease without life-threatening implications, and Class IV indicates life-threatening systemic disorders^[5]^. The World Health Organization classifies adults with a BMI of 25–29.9 kg/m^2^ as overweight and ≥30 kg/m^2^ as obese^[6]^. Large observational cohorts and registry studies have consistently shown that advanced age, higher ASA class, diabetes, immunosuppression, obesity (BMI ≥ 30 kg/m^2^), and prolonged operative time (>60 minutes) significantly increase the risk of SSI in LC^[7]^. Acute or inflammatory cholecystitis with anticipated bile spillage further amplifies infection risk^[8]^. Low-risk patients are therefore those with ASA I–II, BMI < 25 kg/m^2^, absence of significant comorbidities (e.g., no diabetes or immunosuppression), and uncomplicated, elective disease. Moderate-risk patients include ASA III or those with a BMI of 25–29.9 kg/m^2^ or well-controlled comorbidities. High-risk patients comprise ASA IV–V, BMI ≥ 30 kg/m^2^, poorly controlled or multiple comorbidities, or the presence of acute/inflammatory cholecystitis with expected bile spillage. This schema aligns established physiological classifications and empirical SSI risk factors to clarify which patient subsets may or may not benefit from perioperative antibiotic prophylaxis.

**HIGHLIGHTS**

- Routine antibiotics offer minimal benefit in low-risk laparoscopic cholecystectomy.
- Most surgical-site infection reductions involve superficial infections, not deep or organ-space sites.
- Absolute infection risk is low; numbers needed to treat range from 30 to 50 across studies.
- Adverse effects and resistance risks argue against widespread antibiotic use.
- Guidelines recommend selective, not routine, prophylaxis to support stewardship.

In preparation of this article, AI-assisted tools were used in accordance with the TITAN Guidelines 2025^[9]^. Specifically, ChatGPT (OpenAI) was utilized for drafting and refining text, Grammarly was used for grammar and language editing, and Jenny AI supported citation management and reference formatting.

## Guidelines and practice patterns

Leading SSI guidelines classify uncomplicated LC as a clean or clean-contaminated case where prophylaxis is usually not indicated^[2,3]^. The SIGN and ASHP recommendations both state that healthy patients undergoing elective LC do not require perioperative antibiotics^[3]^. These recommendations reflect older meta-analyses and expert consensus, and they emphasize antibiotic stewardship and avoidance of unnecessary drug use. In practice, however, many surgeons still administer antibiotics “just in case.” For example, Vohra *et al* found that 61% of patients undergoing nonemergency LC in a UK multicenter registry received prophylaxis^[4]^. This high usage contrasts with the 1.2–3.2% SSI rates observed when no antibiotics are given^[1]^, and it raises questions about cost, side effects, and resistance. The next sections analyze the evidence from trials, reviews, and meta-analyses to clarify whether routine prophylaxis actually reduces infections.

## Evidence from trials and meta-analyses

Over the past two decades, numerous randomized controlled trials (RCTs) and reviews have directly compared perioperative antibiotics versus none in elective LC. A 2014 multicenter RCT (*n* ≈ 1037) from Japan found significantly fewer infections with prophylaxis: SSI occurred in 0.8% of the antibiotic group versus 3.7% of controls (OR ≈ 0.205, *P* = 0.001)^[10]^. The authors concluded that antibiotics “should be recommended” for LC to prevent infections^[10]^. In contrast, a 2017 Turkish trial reported no difference: total SSI was ~1.2% overall, with 1.5 versus 1.04% in the antibiotic versus no-antibiotic arms (*P* = 1.0), leading to the statement “antibiotic prophylaxis is not needed” for elective LC^[1]^. These single trials illustrate the small absolute event rates: even when differences are significant, only a handful of infections are prevented, and some studies lack the power to detect such differences.

Given conflicting single trials, systematic reviews and meta-analyses have attempted to pool the data. Liang *et al* combined 21 RCTs (*n* ≈ 5207) and found a significant reduction in SSI with antibiotics: risk ratio (RR) 0.61 (95% CI: 0.45–0.82, *P*= 0.001)^[11]^. Similarly, Kim *et al* pooled 28 RCTs plus observational studies (total ~12 121 patients) and reported lower SSI in the prophylaxis group (overall SSI 1.54 vs 2.63%; RR ≈ 0.67, 95% CI: 0.51–0.88, *P*= 0.003)^[3]^. These meta-analyses suggest that antibiotic administration roughly *halves* the relative risk of infection, albeit from a very low baseline. In mathematical terms, numbers needed to treat (NNT) remain large. For instance, the UK CholeS study (matched cohort) found superficial SSI of 0.7% with antibiotics versus 2.3% without, yielding an NNT of ~45^[4]^.

Other reviews have been less convinced. Pasquali *et al* analyzed 19 RCTs (*n* ≈ 5259) and found no significant difference in SSI: 2.4 versus 3.2% (antibiotic vs none; RR ≈ 0.81, 95% CI: 0.58–1.13)^[2]^. They concluded that “antibiotics should not be administered” for low-to-moderate risk cholecystitis patients^[2]^. A Cochrane review (2022) similarly found insufficient evidence of benefit (OR 0.87, 95% CI: 0.49–1.54) and called for more trials^[12]^. In sum, three broad patterns emerge: (1) large trials or pooled analyses often show a statistically significant reduction in infections^[3,10,11]^, but (2) the absolute event rates are very low, yielding modest absolute benefit and lack of significance in some studies^[1,2]^.

The divergent findings can be partly explained by study design and patient selection. Meta-analyses that included all data (even lower-quality non-RCT data) tend to favor antibiotics^[3,13]^, whereas analyses restricted to high-quality RCTs often do not show significant reductions^[2,12]^. Some authors have re-evaluated previous meta-analyses: for example, a 2018 BMJ Open overview re-analyzed published reviews (excluding flawed trials) and found that all pooled measures (SSI, distant infections, overall) were significantly lower with prophylaxis (SSI RR ≈ 0.71, 95% CI: 0.51–0.99)^[13]^. Likewise, Yang *et al* meta-analyzed 33 studies and reported significant effects (e.g., SSI RR ≈ 0.57, 95% CI: 0.33–0.98)^[14]^, concluding that perioperative antibiotics “are effective…in low-risk patients”^[14]^.

These analyses often combine data from heterogeneous settings (biliary colic, acute cholecystitis, pediatrics, etc.), which may limit direct applicability. A key point is that deep/organ-space infections are exceedingly rare in LC; most SSIs are superficial port-site infections^[1,3]^. The meta-analyses consistently show benefit only in superficial SSI rates, with virtually no effect on deep infection^[3]^. They also agree that the length of stay is marginally shorter with antibiotics and that adverse effects are infrequent^[11,15]^. In other words, prophylaxis appears to trim the tail of superficial infections at the expense of antibiotic use.

In Table 1, key studies emphasize that antibiotic prophylaxis can reduce SSI incidence, but only at the cost of treating many patients unnecessarily. Current best practice is to follow guideline recommendations for low-risk cases and reserve antibiotics for cases with added risk factors.

**Table 1:** Key studies of antibiotic prophylaxis in elective LC

Study	Design (*n*)	Antibiotic vs none	SSI (AB vs no AB)	Effect (95% CI)
Matsui *et al* ^[10]^	RCT (*n* = 1037; 518 vs 519)	Cefazolin periop	0.8 vs 3.7%	OR 0.205 (0.069–0.606) (*P* = 0.001)^[10]^
Liang *et al* ^[11]^	Meta (21 RCTs, *n* = 5207)	Various (2–10 doses)	–	RR 0.61 (0.45–0.82) (*P* = 0.001)^[11]^
Kim *et al* ^[3]^	Meta (28 RCTs + 6 non-RCTs, *n* ≈ 12 121)	Various	1.54 vs 2.63%	RR 0.67 (0.51–0.88) (*P* = 0.003)^[3]^
Vohra *et al* ^[4]^	Cohort (matched, *n* = 2538)	Cefuroxime or ceftriaxone	0.7 vs 2.3% (superficial SSI)	*P* = 0.001 (NNT = 45)^[4]^
Sanabria *et al* ^[12]^	Cochrane (11 RCTs, *n* = 1664)	Various	–	OR 0.87 (0.49–1.54)^[12]^ (not significant)

OR, odds ratio; RR, risk ratio; NNT, number needed to treat.

Summary of representative trials and reviews comparing perioperative antibiotics vs no antibiotics in elective LC.

## Discussion

### Weighing benefits versus stewardship

The data suggest that prophylactic antibiotics can reduce superficial wound infections in LC, but the absolute benefit is small. Most reported NNTs exceed 30–50^[4,10]^, meaning many patients receive antibiotics to prevent a single SSI. Critics point out that SSI in LC is usually mild and treatable, whereas antibiotic use carries risks of allergic reaction, *Clostridioides difficile* infection, and antibiotic resistance. From a public health standpoint, the marginal gain must be balanced against these risks and costs (drug and nursing time)^[2,13]^. Indeed, proponents of guideline recommendations emphasize that routine prophylaxis for low-risk patients is not “worth it” given the low infection rates^[2]^.

On the other hand, some circumstances may justify prophylaxis. For example, intra-operative spillage of bile or pus, prolonged surgery time, or institutional norms may tilt the balance. The BJS meta-analysis by Pasquali *et al* included many cases of low–to–moderate risk cholecystitis (Tokyo grade I–II) and still found no SSI benefit^[2]^. By contrast, Japanese and some European surgeons may consider antibiotics in any LC with acute cholecystitis. Importantly, the evidence we reviewed applies to *elective*, generally healthy patients. In acute/inflammatory cases, separate guidelines exist. For example, in mild acute cholecystitis, certain studies suggest perioperative antibiotics may be reasonable, whereas in severe cases, preoperative antibiotics are standard.

At a policy level, antibiotic stewardship committees often recommend *against* routine prophylaxis in low-risk LC, echoing SIGN/ASHP^[2,3]^. This is to avoid contributing to resistance. However, some recent editorials and reviews have challenged this dogma, citing meta-analytic evidence of benefit^[13,14]^. In settings with high baseline SSI or resistant flora, the calculus may change. Moreover, patient-level factors (ASA class, diabetes, and obesity) deserve consideration. (Notably, Sarkut *et al* found diabetes and obesity *did not* affect SSI risk in their series^[1]^, but this finding is not universal.)

### Limitation

While routine practices surrounding prophylactic antibiotic use in elective LC are well established in many developed healthcare systems, the relevance of this review remains significant for developing regions. In settings lacking standardized surgical protocols or stewardship oversight, synthesis of high-quality evidence can guide context-specific implementation. Additionally, the evolving landscape of antimicrobial resistance underscores the global importance of revisiting even established practices to support evidence-based decision-making.

## Conclusion

In summary, *routine* perioperative antibiotics for low-risk elective LC are not strongly supported by most guidelines, which reflect historical evidence and stewardship goals^[2,3]^. Recent high-profile RCTs and meta-analyses show that antibiotics can statistically reduce superficial infection rates^[3,10,11]^, but the clinical significance is modest given the low baseline SSI risk. Opinion varies: some experts argue the modest reduction justifies prophylaxis, while others emphasize unnecessary antibiotic use. Ultimately, decision-making should be individualized. If prophylaxis is used, evidence suggests multiple-dose regimens are more effective than single-dose^[11]^. Otherwise, strict sterile technique and rapid conversion to open surgery when indicated are key. Given the evolving evidence, further large-scale trials (or high-quality registries) could clarify subgroups that benefit most. Until then, surgeons should discuss the pros and cons with patients and tailor prophylaxis to each clinical context.

## Data Availability

All data generated during this study are included in this published article.
